# 2′‐Fucosyllactose Modulates the Intestinal Immune Response to Gut Microbiota and Buffers Experimental Colitis in Mice: An Integrating Investigation of Colonic Proteomics and Gut Microbiota Analysis

**DOI:** 10.1002/fsn3.70418

**Published:** 2025-06-10

**Authors:** Jiamin Dong, Minyan Qian, Dong Zhou, Aoshuang Zhu, Wenting Zhang

**Affiliations:** ^1^ Central Laboratory of Pediatrics Changzhou Children's Hospital Affiliated to Nantong University Changzhou China; ^2^ Pharmaceutical Laboratory Asthma and Bronchitis Research Center of Changzhou Changzhou China

**Keywords:** 2′‐fucosyllactose, gut microbiota, inflammatory bowel disease, proteomics

## Abstract

Inflammatory bowel disease (IBD), a recurrent gastrointestinal disease, is characterized by dysbiosis and inflammation. 2′‐fucosyllactose (2′‐FL) shows benefits in maintaining gut microenvironment homeostasis. This study aims to investigate the protective mechanism of 2′‐FL against experimental IBD using integrated analysis of colonic proteomics and microbiota 16S rRNA sequencing technologies. A murine model of experimental colitis was induced by dextran sodium sulfate (DSS) for 7 days (day 0‐day 7). 2′‐FL (250 mg/kg/d) was orally administered for 7 days. On day 7, all mice were sacrificed and their colon tissues were collected for morphological examination and label‐free quantitative proteomics analysis. The fecal samples were collected for microbiota 16S rRNA sequencing. 2′‐FL significantly ameliorated DSS‐induced pathological damage and restored the epithelial integrity of colon tissues in mice. Colonic proteomics and GO analysis showed 681 differentially expressed proteins (DEPs) in response to 2′‐FL administration. These DEPs were mainly enriched to GO terms of *response to bacterium* and *acute‐phase response*. In addition, 2′‐FL reversed the compensatory increase of haptoglobin, serpina3n, Arg2 and Reg3. It also reduced the accumulation of pro‐oxidant indicator Cyp2e1, in the colon of colitis mice. Moreover, 2′‐FL ameliorated colonic dysbiosis by suppressing the overgrowth of pathogenic *Proteobacteria* and reversed the reduction in abundance of prebiotic bacterial genus including *Lachnospiraceae_NK4A136_group*. Notably, the expression of Arg2 and Cyp2e1 showed strong correlation; Reg3b and Reg3g were significantly associated with *Lactobacillus*. 2′‐FL prevented DSS‐induced intestinal inflammatory damage through exerting prebiotic capacity and regulating the intestinal immune response to gut microbiota.

## Graphical abstract

2′‐fucosyllactose prevented DSS‐induced intestinal inflammatory damage through exerting prebiotic capacity and regulating the intestinal immune response to gut microbiota.

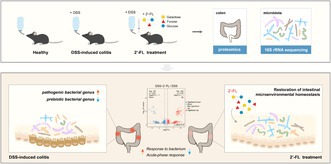

## 1 Introduction

Inflammatory bowel disease (IBD), primarily encompassing ulcerative colitis (UC) and Crohn's disease (CD), is a recurrent gastrointestinal disorder characterized by immune dysregulation and inflammation that can affect the entire gastrointestinal tract. Its pathogenesis is closely linked to genetic predisposition, environmental factors, and immune dysfunction (Bedke et al. 2024; Qiu et al. 2022). In recent years, the incidence and prevalence of IBD have risen significantly, especially in children and adolescents, posing a growing public health concern (Foppa et al. 2024). Pediatric IBD often presents with more extensive phenotypic manifestations compared to adult‐onset IBD, and is frequently cause of growth impairment, delayed puberty, depression, and anxiety (Agrawal et al. 2021; Otaru et al. 2024; Stapersma et al. 2018). There is an urgent need to develop effective preventive strategies and identify precise therapeutic targets to mitigate the burden of IBD.

The etiology of IBD remains elusive, and emerging evidence highlights the critical role of the gut microbiota in the pathogenesis of IBD, alongside genetic, environmental, and immunological factors (Li et al. 2020). The gut microbiota, characterized by its vast diversity and high density, plays a pivotal role in regulating host intestinal metabolism and immune homeostasis through its involvement in nutrient metabolism, drug disposal, and the production of antimicrobial metabolites. Under healthy physiological conditions, the gut microbiota maintains a balanced state. However, disruptions in this equilibrium, termed dysbiosis, lead to a dysfunctional intestinal microenvironment and are involved in the development of IBD. In IBD, dysbiosis is marked by a significant diminishment in microbial diversity, along with an imbalance between pathogenic and beneficial bacteria. IBD leads to a decreased abundance of anti‐inflammatory bacteria such as *Faecalibacterium* and the overgrowth of certain anaerobic bacteria such as *Ruminococcus gnavus* and *Fusobacterium species* (Hua et al. 2023; Ventin‐Holmberg et al. 2022; Zuo and Ng 2018). Moreover, the gut microbiota functions as producers of bioactive metabolites, such as short‐chain fatty acids (SCFAs), bile acids (BAs), and tryptophan metabolites, which are essential for maintaining intestinal homeostasis and modulating immune responses (Ghosh et al. 2021; Lavelle and Sokol 2020; Liu et al. 2022). IBD patients exhibit abnormal production of these small molecular metabolites. It is known that IBD leads to a significant reduction in the level of acetate, a key SCFA involved in regulating intestinal pH and preserving mucosal integrity (Jagt et al. 2022). Also, IBD increases the level of primary bile acid and reduces the level of secondary bile acid concentrations (Pan et al. 2024). The dysbiosis can compromise intestinal barrier function, either through the production of harmful metabolites or direct microbial invasion, leading to increased intestinal permeability, dysregulated immune responses, and the consequent onset of IBD. Strategies targeting the gut microbiota along with its metabolites represent a promising approach for the prevention and treatment of IBD.

Human milk oligosaccharides (HMOs), a group of highly abundant and structurally diverse prebiotics in breast milk, are composed of D‐galactose (Gal), D‐glucose (Glc), N‐acetylglucosamine (GlcNAc), sialic acid (SA), and L‐fucose (Fuc). They play a multifaceted role in promoting digestive and immune system maturation, as well as supporting neuro‐cognitive development (Dinleyici et al. 2023; Hill et al. 2021), which has been previously found in infantile and pediatric diseases. Structurally, HMOs can be classified into three categories: neutral HMOs without fucosylation, neutral HMOs with fucosylation (Fuc‐HMOs), and acidic HMOs with sialylation (Sia‐HMOs) (Moubareck 2021). 2′‐fucosyllactose (2′‐FL), the most abundant HMO, has demonstrated significant therapeutic potential in experimental necrotizing enterocolitis (NEC), a fatal intestinal emergency in newborns, by enhancing intestinal barrier integrity, alleviating gut dysbiosis, mitigating overactivation of the TLR4/NF‐κb pro‐inflammatory pathway, and improving local micro‐perfusion (Hackam and Sodhi 2022; Zhang et al. 2023). The dyshomeostasis of microbiota and immune system is the common soil in the etiological mechanism of NEC and IBD; it has been previously reported that 2′‐FL showed protection in DSS‐induced experimental colitis (Mortaz et al. 2022; Yao et al. 2022). However, the mechanism of 2′‐FL alleviating IBD symptoms and damage remains elusive.

Proteomics is the precise and comprehensive tool for high‐throughput sequencing of proteins in a specific cell, tissue, or organism (Letunica et al. 2022). By leveraging mass spectrometry (MS)‐based techniques, proteomics separates proteins, determines the amino acid sequences, and protein abundance for analyzing protein structures, protein–protein interactions, function, and the prediction of potential bioactive targets (Mendes and Dittmar 2022; Shuken 2023). Proteomics techniques are primarily subdivided into label‐based quantitative proteomics and label‐free quantitative proteomics. The former is limited by the type and quantity of labels, while the latter has a wider range of applications (Boesl 2017). Moreover, label‐free quantitative proteomics offers several advantages compared with traditional biochemical and molecular biology techniques, including broader applicability, shorter time, higher precision, greater reproducibility, and the ability to generate large‐scale datasets. These features make it an invaluable approach for uncovering novel mechanisms and therapeutic targets in complex diseases such as IBD.

In this study, we aim to elucidate the mechanisms by which 2′‐FL alleviates intestinal inflammation using a combination of colonic proteomics and microbiota 16S rRNA sequencing in a murine model of DSS‐induced IBD. We investigated and validated the protection of 2′‐FL against intestinal inflammation and damage in IBD mice, and subsequently identified differentially expressed proteins and microbial taxa associated with functional pathways following 2′‐FL administration. Our findings provide new insights into the prebiotic action, protective effects, and potential mechanisms of 2′‐FL against IBD.

## 2 Materials and Methods

### 2.1 Animal Experiment

Six‐week‐old female C57BL/6 mice were kept in a 12:12 h dark/light cycle within a specific pathogen‐free animal breeding environment that conformed to the guidelines described in the revised Regulations for the Administration of Affairs Concerning Experimental Animals 2017 in China. All animals were allowed to survive with free access to diet and water. The experimental protocols involving animals were conducted with the approval of the Ethical Committee of Changzhou Children's Hospital Affiliated to Nantong University (Changzhou, China) (Approval Number: 2023‐011).

All the mice were divided into three groups using a randomness strategy: control group, DSS group, DSS + 2′‐FL group. Experimental colitis in mice was induced by unrestricted access to 3% (wt/vol) DSS (MP Biomedicals, CAS Number: 9011‐18‐1, molecular weight of 36,000–50,000) in drinking water for a 7‐day period, while the parallel control group had distilled water (Mo et al. 2022). The DSS + 2′‐FL group had unlimited access to 3% DSS water and was gavaged with an additional 2′‐FL (250 mg/kg) (CAS: 41263‐94‐9) per day. 2′‐FL (purity: 95%) was gifted by the laboratory of Professor Hongtao Zhang (Jiangnan University, Wuxi, China). The mice were quickly decapitated to collect cecal feces and colon tissue after being anesthetized using 0.3% pentobarbital sodium through intraperitoneal injection. The data of disease activity index (DAI) and colonic length were recorded daily, and the cecal feces and colon tissues of mice were quickly harvested and stored at −80°C at the end of the experiment.

### 2.2 Assessment of Disease Activity Index (DAI) Score Assessing Disease Severity

To assess the severity of colitis, the body weight, stool condition, and blood in the stool were determined according to the published grading standard (Kim et al. 2023; Liu et al. 2021). Weight loss was scored as follows: score 0, none; score 1, 1%–5%; score 2, 5%–10%; score 3, 10%–20%; score 4, > 20%. Stool condition was scored as follows: score 0, normal; score 2, loose stools; score 4, watery diarrhea. Blood in stool was scored as follows: score 0, normal; score 4, gross bleeding.

### 2.3 Hematoxylin‐Eosin (H&E) Staining

The colon tissues were fixed in 4% formaldehyde for 1 day and subsequently dehydrated by sequential immersion in ethanol solutions of different concentrations (75%, 85%, 95%, and 100%) for 30 min. The dehydrated colon tissues were immersed in a mixture of anhydrous ethanol and xylene for 1 h. After immersing in xylene twice (1 h each time), the colon tissue was immersed in paraffin wax three times (1 h each time). The colon tissue was wrapped in heated paraffin and then cooled at 20°C for 20 min. The cured tissues were cut into 4 μm slices. The slides were dried at 60°C for 24 h on glass slides. The sections of colon tissue were deparaffinized and hydrated in different concentrations (100%, 90%, 75%) of xylene and ethanol. The sections of colon tissue were stained with hematoxylin and eosin (H&E) and evaluated using a light microscope. The pathology scores were based on references (Liu et al. 2022).

### 2.4 Proteomic Analysis and Bioinformatics Analysis

The colon tissues were collected for label‐free quantitative proteomics. Total protein was extracted and the concentration of total proteins was determined via BCA quantification kit. Peptide fragments were desalted and dried, following the digestion of total proteins treated by trypsin. Equal quantities of peptides were taken for each sample and separated using EASY‐nLC 1200 liquid phase, with mobile phase A being 0.1% FA aqueous solution and mobile phase B being 0.1% FA in ACN. The liquid gradient was set to the following: 0–20 min, 5%–22% B; 20–24 min, 22%–37% B; 24–27 min, 37%–80% B; 27–30 min, 80% B. Peptides were separated by an ultra‐high performance liquid chromatography system and injected into a timsTOF Pro mass spectrometer (Bruker) for analysis. DIA raw data were analyzed using Spectronaut Pulsar 18.4 (Biognosys, Switzerland). Differentially expressed proteins were required to fulfill *p* < 0.05 and fold change (FC) ≥ 1.2 or FC ≤ 0.67. Differentially expressed proteins were regarded as significantly upregulated proteins when *p* < 0.05 and FC ≥ 1.2, while they were regarded as significantly down‐regulated proteins when *p* < 0.05 and FC ≤ 0.67. The Gene Ontology (GO) was applied to analyze the biological process (BP), cellular component (CC) and molecular function (MF) of the differentially expressed proteins according to their biological functions and classification. Differential protein interactions (PPI) were analyzed based on the string database, and differential protein interaction networks were constructed. Gene set enrichment analysis (GSEA) was performed using GSEA software (version 4.1.0). The correlation heatmap of interrelationships among differentially expressed proteins was analyzed by Spearman correlation coefficients.

### 2.5 Microbial 16S rRNA Sequencing and Analysis

The fecal samples were collected for label‐free quantitative proteomics. The total genomic DNA of the microbial community was extracted from each group of fecal samples (FastPure Stool DNA Isolation Kit; MJYH, Shanghai, China). The extracted genomic DNA was detected by using electrophoresis (1% gel), and the DNA concentration and purity were determined by NanoDrop2000 (Thermo Scientific, USA). PCR was performed on the V3–V4 variable region of the 16S rRNA. Products of PCR were recovered using a 2% gel and purified by PCR Clean‐Up Kit (China). cDNA library was constructed from purified PCR products using the NEXTFLEX Rapid DNA‐Seq Kit. Sequencing was carried out on the Illumina Nextseq 2000 platform. Operational Taxonomic Units (OTUs) were clustered based on 97% similarity. Species taxonomy of OTUs was annotated using the RDP classifier (http://rdp.cme.msu.edu/, version 2.11) for comparison with the Silva 16S rRNA database (v138). The community composition of each group was assessed at different species classification levels. Alpha diversity such as Ace, Shannon, Sobs, and Chao indices was calculated by mothur (http://www.mothur.org/wiki/Calculators). Difference in alpha diversity between groups was assessed by the Wilcoxon rank‐sum test. The similarity of microbial community structure among groups was assessed by NMDS and PCA. NMDS was based on the Hellinger distance algorithm. The correlation heatmap of interrelationships between differentially expressed proteins and differential bacterial genera was performed using Spearman correlation coefficients.

### 2.6 Statistics Analysis

The data expressed as mean ± SD were analyzed by GraphPad Prism 7.0. To test differences among and between groups, one‐way ANOVA, Kruskal‐Wallis test, and Tukey–Kramer comparison were used. *p* < 0.05 was considered statistically significant.

## 3 Results

### 3.1 Effects of 2′‐FL on Disease Activities and Histo‐Morphological Damage in Mice With DSS‐Induced Colitis

**FIGURE 1:**
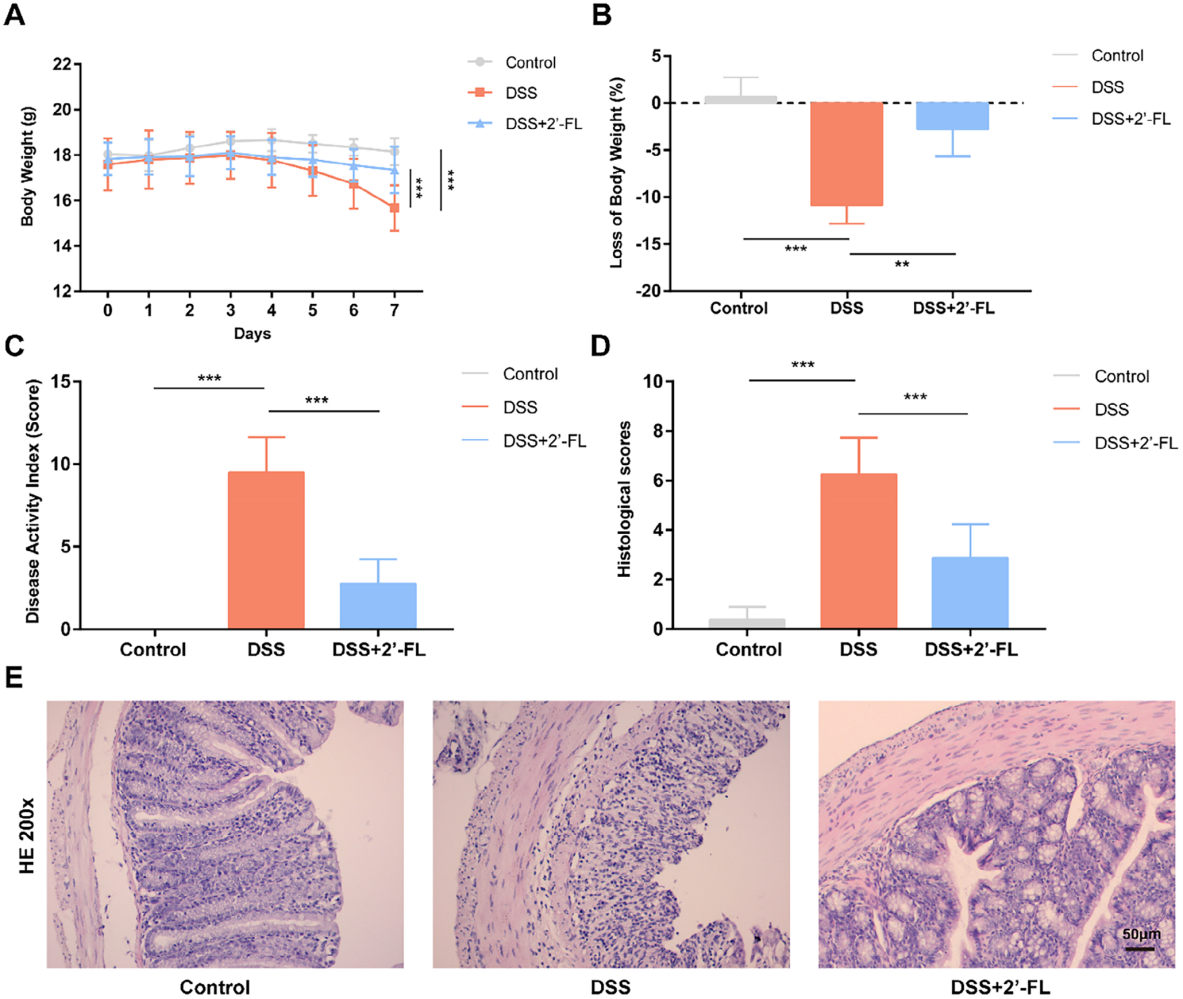
2′‐FL alleviated the disease activity and pathological lesion in DSS‐induced colitis in mice. (A) Body weight change curve, (B) loss of body weight, (C) disease activity index score, and (D, E) representative H&E staining images and scores of colon pathologic damages on day 7. Data were expressed as the mean ± SD (*n* = 8). **p < 0.01, ***p < 0.001.

To verify whether 2′‐FL could effectively mitigate intestinal inflammation, the acute colitis model in mice was induced by DSS. A significant decrease in body weight of the DSS group was observed over time (Figure 1A), starting to lose weight on day 5, and the body weight had significantly reduced (−10.85%, *p* < 0.05) at the end of the experiment after DSS treatment (Figure 1B). DAI score (9.5, *p* < 0.001) was increased in DSS‐treated mice compared with the control group (Figure 1C). After administration of 2′‐FL, the body weight loss of mice was observably attenuated (Figure 1B) and DAI scores were markedly decreased (Figure 1C) compared to the DSS group. In addition, hematoxylin‐eosin staining (H&E) of colon tissues collected from mice suggested inflammatory colonic lesions such as epithelial cell damage, crypt deformity, and inflammatory immune cell infiltration in DSS‐induced colitis, which was alleviated by 2′‐FL (Figure 1D,E). It was suggested that 2′‐FL effectively alleviated DSS‐caused inflammatory disease activity and histomorphological damage in mice with DSS‐induced acute colitis.

### 3.2 2′‐FL Modulates the Colonic Proteomics Profile in DSS‐Treated Mice

**FIGURE 2:**
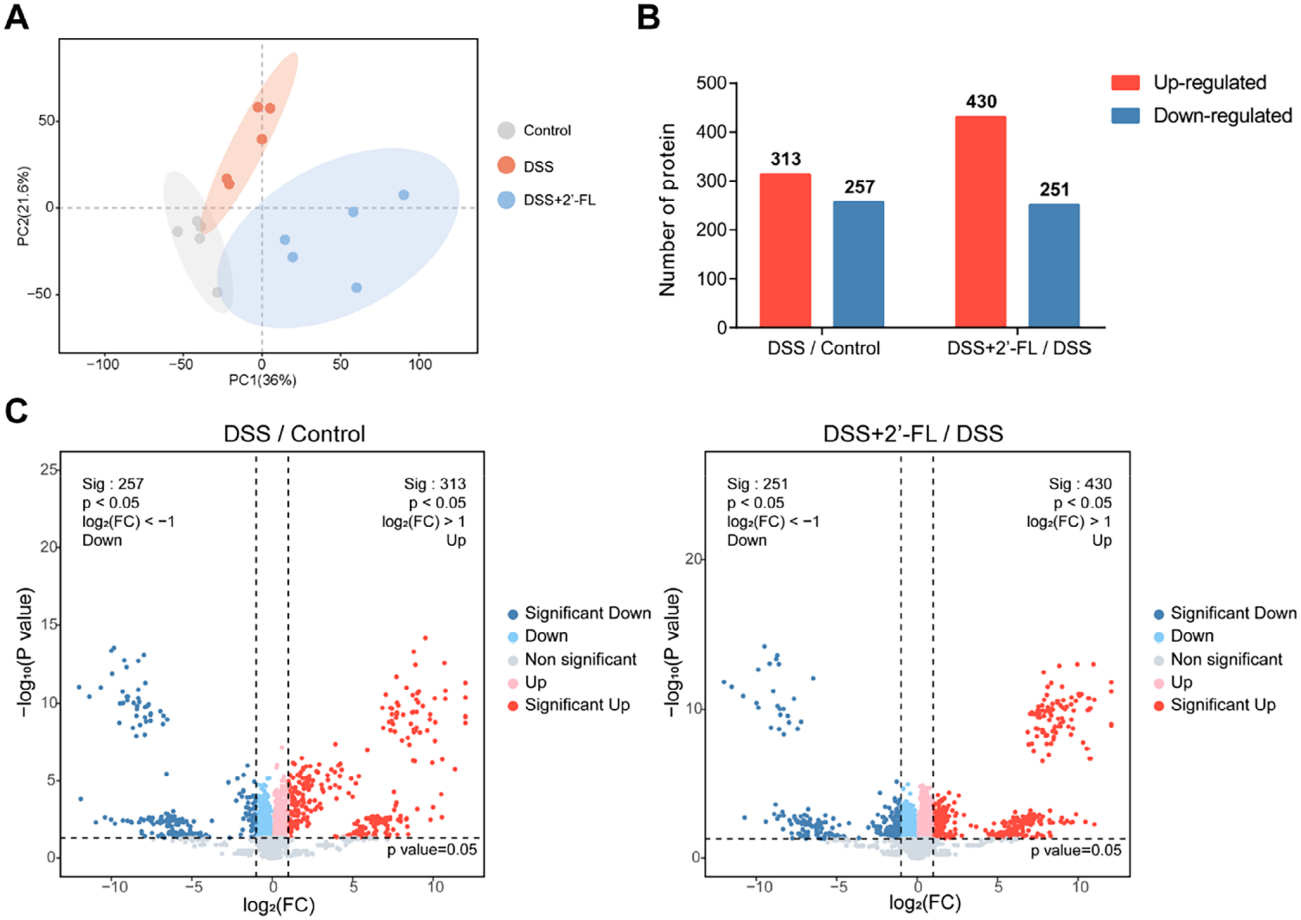
Principal component analysis (PCA) and volcano plot of DEPs in response to DSS and 2′‐FL. (A) PCA of proteomics in the colon samples from control group, DSS group, and DSS + 2′‐FL group; (B, C) number of DEPs between DSS group and control group, and between DSS + 2′‐FL group and DSS group (*n* = 5).

Colon samples from the control group, the DSS group, and the DSS + 2′‐FL group were analyzed by label‐free quantitative proteomics. The proteomic profiling of colon tissues from three experimental groups was distinguished (Figure 2A). The volcano plot exhibited numbers of differentially expressed proteins (DEPs) across the experimental groups (Figure 2B–D). The DSS group demonstrated a marked reduction in 257 proteins alongside a substantial upregulation of 313 proteins compared to the control group. The comparison between the DSS groups and the DSS + 2′‐FL group showed more DEPs, with 251 proteins exhibiting downregulation and 430 proteins displaying upregulation.

**FIGURE 3:**
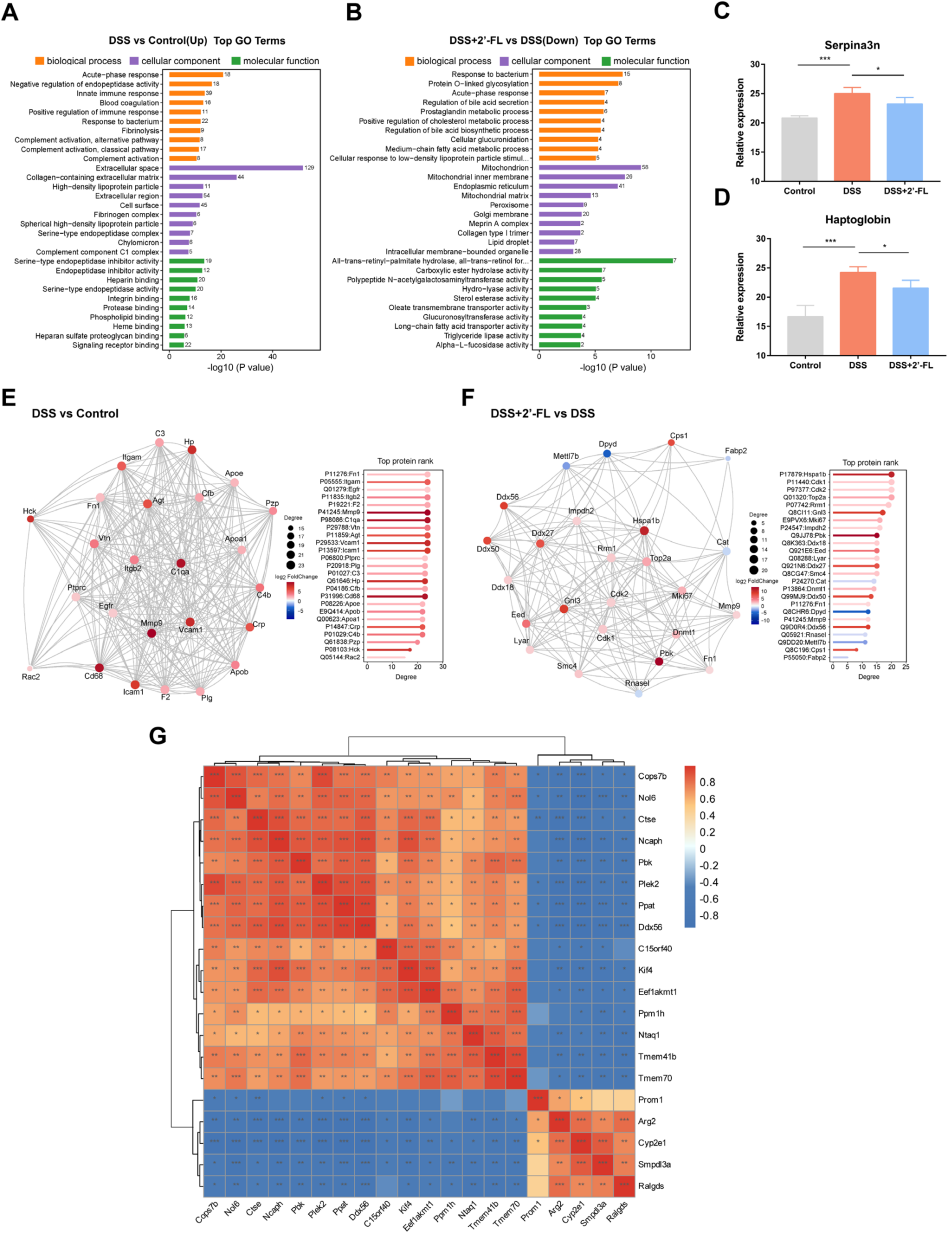
GO analysis and protein–protein interaction (PPI) of DEPs after 2′‐FL administration in mice with DSS‐induced colitis. (A, B) GO analysis of the DEPs between groups; (C, D) expression of serpina3n and haptoglobin, the representative DEPs, in three experimental groups; (E, F) PPI networks of DEPs between groups; (G) correlation heatmap of interrelationships among DEPs. Scale indicates the level of positive (red) or negative (blue) correlation, and asterisks indicate significance. The experimental data were performed by the Spearman correlation coefficients. (*n* = 5) *p < 0.05, **p < 0.01, ***p < 0.001.

On the basis of the analysis of DEPs, DSS induced inflammatory response to bacterium, which showed that its associated differentially upregulated proteins were mainly enriched in the pathways of *signaling receptor binding*, *acute‐phase response*, *response to bacterium*, and *innate immune response* (Figure 3A). In contrast, compared with the DSS group, the relevant differentially down‐regulated proteins in the DSS + 2′‐FL group were mainly enriched in the pathways of *response to bacterium*, *acute‐phase response*, *long‐chain fatty acid transporter activity*, and other pathways (Figure 3B). The GO enrichment showed that these preponderances of DEPs are all closely associated with intestinal microbiota and inflammatory response, such as haptoglobin and serpina3n (Figure 3C,D). Also, results from the top25 connectivity protein interactions network demonstrated the relevance of representative DEPs (Figure 3E,F). On the basis of the protein levels in the proteomic analysis, significantly upregulated/downregulated DEPs were screened out (Table S1). The overexpression of arginase‐2 (Arg2) (a regulatory protein of inflammatory mediators), cytochrome P4502E1 (Cyp2e1) (one of the oxidative stress indicators), serum amyloid A3 (Saa3) (a pro‐inflammatory cytokine), serpina3n (an acute‐phase inflammatory protein), and haptoglobin (an acute‐phase antibacterial protein) in mice with DSS‐induced colitis were significantly reduced after 2′‐FL administration. In addition, 2′‐FL significantly rescued the abnormal downregulation of growth‐regulatory proteins cathepsin E (Ctse), lymphokine‐activated killer T‐cell‐originated protein kinase (Pbk), and chromosome‐associated kinesin KIF4 (Kif4) in DSS‐induced colitis mice. Also, to explore the potential regulations and associations of these DEPs, the correlation analysis of top 20 DEPs according to fold changes of protein levels from both comparisons (DSS vs. control and DSS + 2′‐FL vs. DSS) was performed and indicated by Spearman's correlation coefficients (Figure 3G). The expression of Arg2 showed a significant association with that of Cyp2e1, while the four proteins critically involved in the biological processes of metabolism or proliferation, DEAD box protein 56 (Ddx56), condensin complex subunit 2 (Ncaph), Pbk, and amidophosphoribosyltransferase (Ppat), showed relatively high correlations between each other.

**FIGURE 4:**
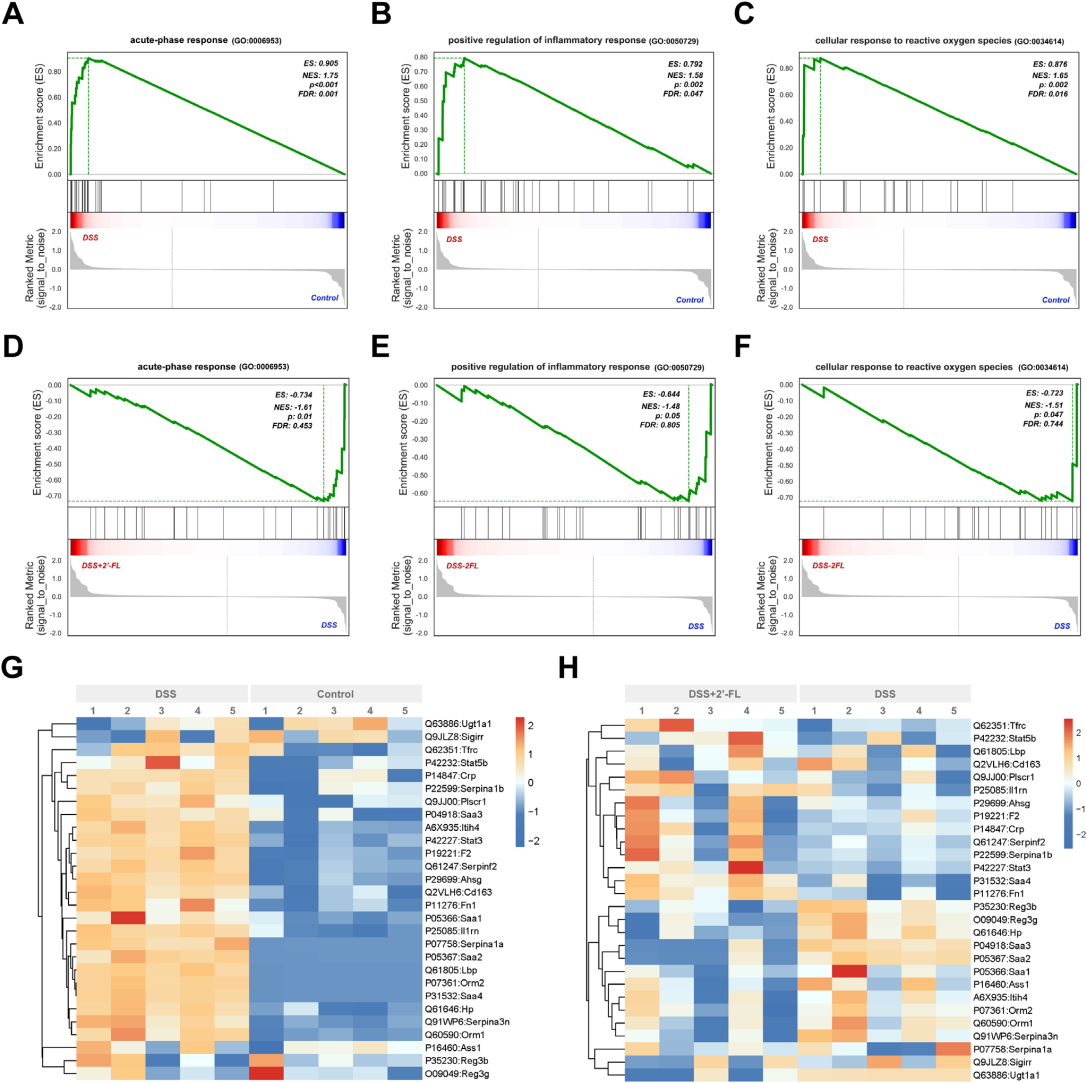
GSEA plot of genes in *acute‐phase response*, *positive regulation of inflammatory response* and *cellular response to reactive oxygen species* gene sets according to their ranked position within the list of DSS or 2′‐FL‐modified proteins. GSEA was probed for the enrichment of the DEPs in *acute‐phase response* (A, D), *positive regulation of inflammatory response* (B, E), and *cellular response to reactive oxygen species* (C, F) gene sets enriched between control group and DSS group, as well as the enrichment of DEGs between the DSS group and DSS + 2′‐FL group. The expression heatmap of DEPs in the GSEA gene set of *acute‐phase response* (G, H) (*n* = 5).

Moreover, gene set enrichment analysis (GSEA) was performed to additionally explore latent critical biological processes in response to the condition of acute colitis and administration of 2′‐FL. GSEA revealed significant enrichment of biological pathways in the DSS group associated with *acute‐phase response* (NES = 1.75, *p* < 0.001), *positive regulation of inflammatory response* (NES = 1.58; *p* = 0.002), *cellular response to reactive oxygen species* (NES = 1.65; *p* = 0.002), *innate immune response* (NES = 1.92; *p* < 0.001), *response to lipopolysaccharide* (NES = 1.75; *p* < 0.001), and *collagen‐containing extracellular matrix* (NES = 1.94; *p* < 0.001) compared to the control group. After 2′‐FL administration, DEPs exhibited distinct molecular signatures dominated by pathways linked with *acute‐phase response* (NES = −1.61, *p* = 0.01), *positive regulation of inflammatory response* (NES = −1.48; *p* = 0.05), *cellular response to reactive oxygen species* (NES = −1.51; *p* = 0.047), *transmembrane transporter activity* (NES = −1.75; *p* < 0.001) and *lipid catabolic process* (NES = −1.67; *p* = 0.003) in DSS‐induced colitis mice (Figure 4A–C).

Notably, pathways related to *acute‐phase response*, *positive regulation of inflammatory response*, and *cellular response to reactive oxygen species* were prominently upregulated in the DSS group compared to controls (Figure 4A–C), while they were down‐regulated in the DSS + 2′‐FL group compared to the DSS group (Figure 4D–F). Also, the expression heatmap in the GSEA gene set of *acute‐phase response* showed the expressive difference of DEPs in the DSS group compared to controls, as well as the DSS + 2′‐FL group (Figure 4G,H).

### 3.3 2′‐FL Regulates the Diversity and Composition of the Gut Microbiota in DSS Mice

Proteomics analysis indicated haptoglobin and serpina3n as critical DEPs involved in tissue protection by 2′‐FL, as their expression showed significant and opposite change in both comparisons (DSS vs. control; DSS + 2′‐FL vs. DSS). Haptoglobin and serpina3n are proteins critical in host immune activity in response to bacterium. In this line, it is possible that gut microbiota is the linchpin for 2′‐FL protecting colon tissue in the condition of DSS colitis. In order to reveal the critically differential intestinal microbiota affected by 2′‐FL, fecal samples from mice were sequenced by 16S rRNA sequencing to investigate the effects of 2′‐FL on the diversity and composition of the gastrointestinal microbiota.

**FIGURE 5:**
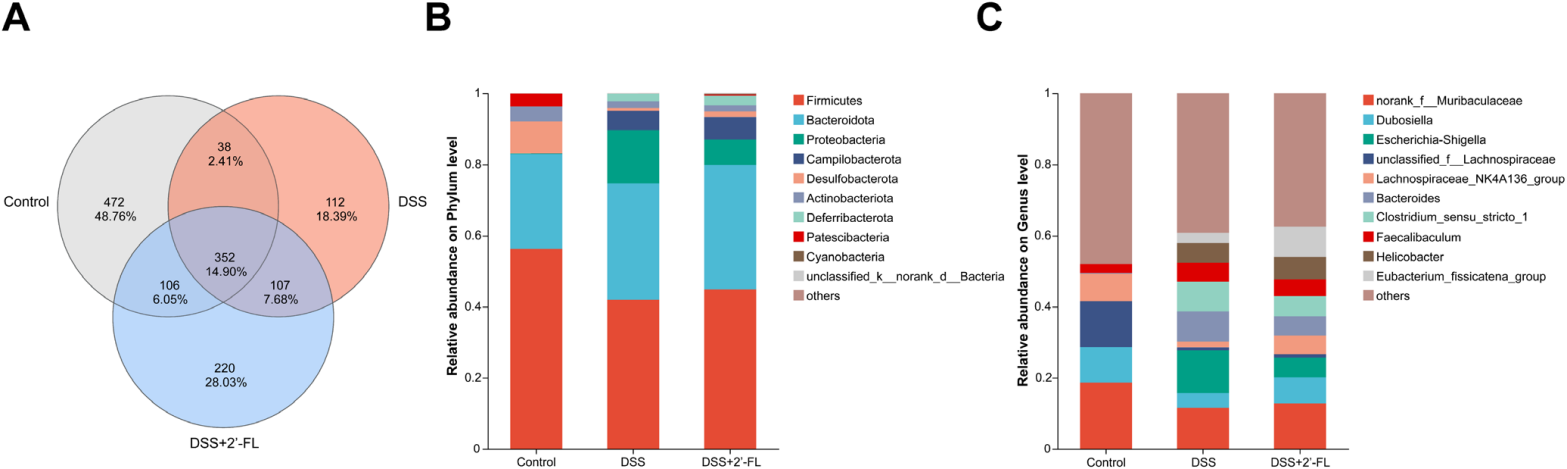
Effects of 2′‐FL on the composition of gut microbiota in DSS mice. (A) Species annotation‐Venn diagram in three groups; (B, C) Composition of gut microbiota from mice at the phylum and genus levels (*n* = 8).

Venn diagram of cecum microbiota showed that a total of 1407 OTUs were detected in all the groups, of which 968, 609, and 785 OTUs were found in the control group, the DSS group, and the DSS + 2′‐FL group, respectively. The control group has the most unique OTUs, with 472 unique OTUs, while the DSS group and the DSS + 2′‐FL group have 112 and 220 unique OTUs. Also, the control group has the highest number of total OTUs and unique OTUs, and the DSS group has the lowest number of both. In addition, there were 390 OTUs overlapping between the control group and the DSS group, 459 OTUs between the DSS group and the DSS + 2′‐FL group, and a total of 352 OTUs overlapping among three groups (Figure 5A).

For the species compositional analysis of the cecum microbiota, *Firmicutes* and *Bacteroidota* were the two most common phyla, accounting for about 70%–80% of the total abundance of intestinal microbiota (Figure 5B,C). At the phylum level, the abundance of the top five microbiota in the control group were *Firmicutes*, *Bacteroidota*, *Desulfobacterota*, *Actinobacteriota*, and *Patescibacteria*, which in the DSS group were *Firmicutes*, *Bacteroidota*, *Proteobacteria*, *Campilobacteriota*, and *Deferribacterota*. The abundance of the top five microbiota in the DSS + 2′‐FL group were the same as those within the DSS group. At the genus level, the abundance of the top five microbiota within the control group were *norank_f__Muribaculaceae*, *unclassified_f__Lachnospiraceae*, *Dubosiella*, *Lachnospiraceae_NK4A136_group*, and *Faecalibaculum*, while in the DSS group were *Escherichia‐shigella*, *norank_f__Muribaculaceae*, *Bacteroides*, *Clostridium_sensu_stricto_1*, and *Helicobacter*. The top five abundances of the DSS + 2′‐FL group were *norank_f__Muribaculaceae*, *Eubacterium_fissicatena_group*, *Dubosiella*, *Helicobacter*, and *Clostridium_sensu_stricto_1*.

**FIGURE 6:**
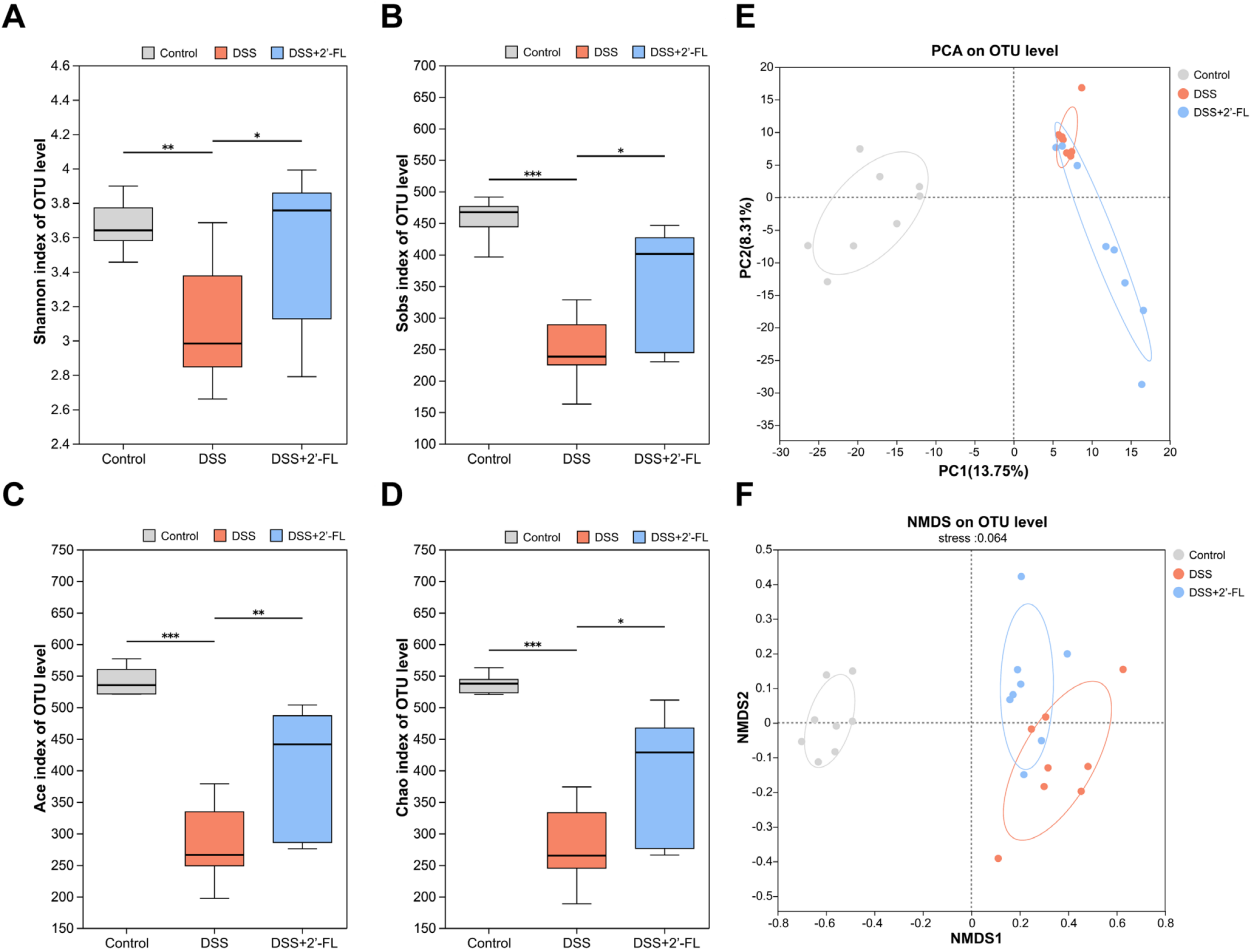
Effect of 2′‐FL on the diversity of gut microbiota in mice with acute colitis. (A) Shannon index, (B) Sobs index, (C) Ace index, (D) Chao index, and β diversity indicated by (E) PCA and (F) NMDS analysis of gut microbiota in three groups (*n* = 8). *p < 0.05, **p < 0.01, ***p < 0.001.

When referring to the diversity and abundance of species, α‐diversity analysis and β‐diversity analysis are commonly used, where α‐diversity includes indices such as Shannon, Sobs, Ace, and Chao, while β‐diversity often uses the principal component analysis (PCA) and non‐metric multidimensional scaling (NMDS) indices. Four α‐diversity analyses were performed. It revealed that the cecum microbiota diversity in the DSS group was significantly decreased compared with the control group, whereas there was markedly increased diversity in the microbiota after administration of 2′‐FL (Figure 6A–D). NMDS analysis was performed based on Hellinger distance. PCA and NMDS analysis further showed that the control group was furthest from the DSS group while the DSS + 2′‐FL group was closer to the control group on the *X* axis (Figure 6E,F). It was indicated that DSS‐induced intestinal inflammation caused a reduction in diversity and dysbiosis in the gut microbiota, which was attenuated by 2′‐FL.

**FIGURE 7:**
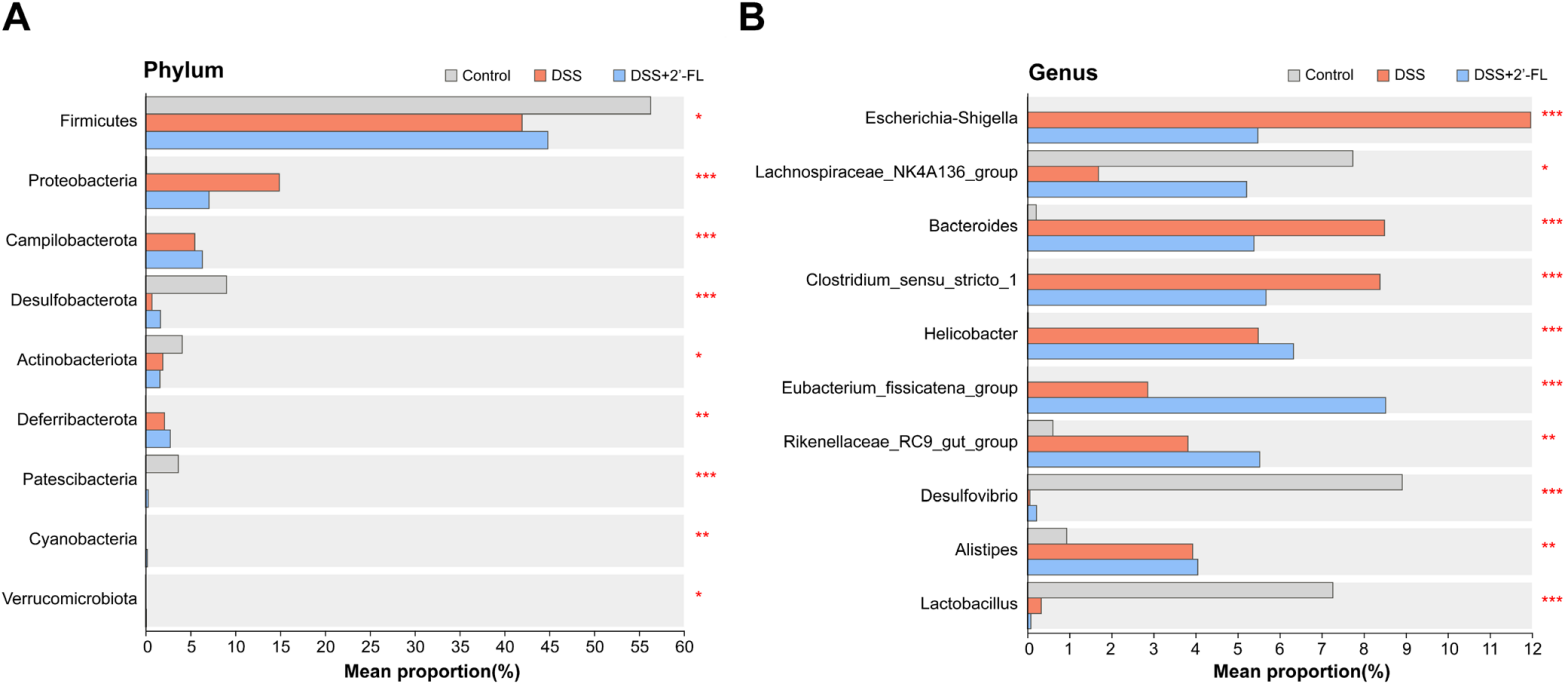
Differential gut microbiota in the DSS‐treated mice with or without 2′‐FL administration. Intergroup significantly differential flora at the phylum level (A) and genus level (B) among groups (*n* = 8). *p < 0.05, **p < 0.01, ***p < 0.001.

In addition, differential microbiota analysis was performed. At the phylum level, DSS‐induced intestinal inflammation caused a significant increase in the relative abundance of *Proteobacteria*, *Campilobacterota*, and *Deferribacterota* compared to the control group, while the relative abundance of *Fimicutes*, *Desulfobacterota*, *Actinobacteriota*, and *Patescibacteria* was decreased. In contrast, the relative abundance of *Proteobacteria* was reversed by 2′‐FL administration and its relative abundance was significantly reduced (Figure 7A). At the genus level, compared to the control group, the relative abundance of *Escherichia‐shigella*, *Bacteroides*, *Clostridium_sensu_stricto_1*, *Helicobacter*, *Eubacterium_fissicatena_group*, *Rikenellaceae_RC9_gut_group*, and *Alistipes* in the DSS group showed a significant increase, with the down‐regulation of abundance in *Lactobacillus, Lachnospiraceae_NK4A136_group*, and *Desulfovibrio*. After 2′‐FL administration, the abundance of *Escherichia‐shigella*, *Clostridium_sensu_stricto_1*, and *Bacteroides* was reduced, along with an upregulation of the abundance of *Desulfovibrio* and *Lachnospiraceae_NK4A136_group* (Figure 7B).

### 3.4 Correlation Between DEPs and Microbiota in the Gut of Mice

**FIGURE 8:**
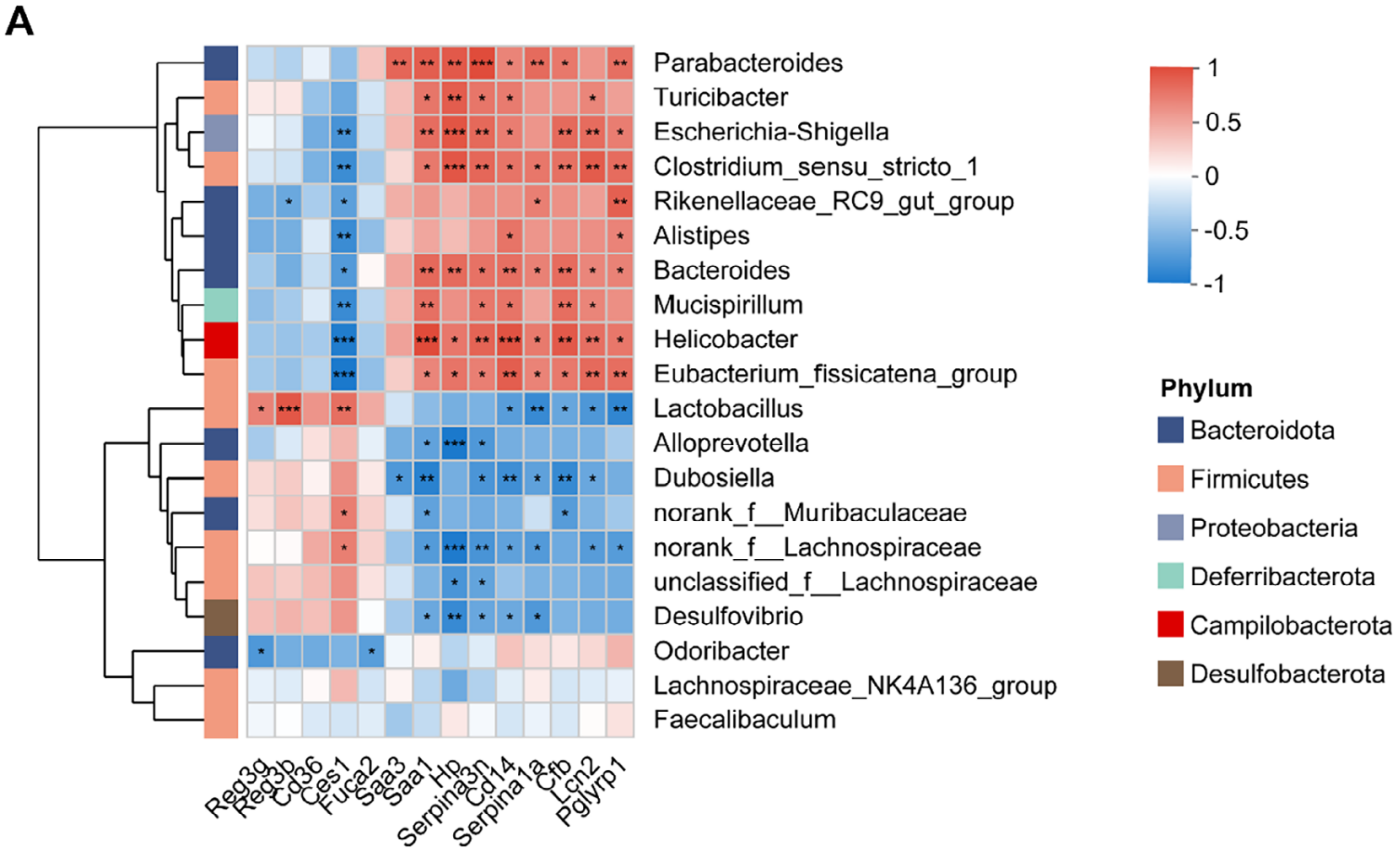
Correlation heatmap of interrelationships between DEPs and differential microbial genera. Scale indicates the level of positive (red) or negative (blue) correlation, and asterisks indicate significance. The experimental data were performed by the Spearman correlation coefficients (*n* = 5). *p < 0.05, **p < 0.01, ***p < 0.001.

In order to explore the critical protein molecules associated with microbial genera involved in 2′‐FL exerting prebiotic and protective effects in DSS colitis, the correlations between DEPs, selected from the GO pathways of *acute‐phase response* as well as *response to bacterium*, and differential bacterial genera were performed by Spearman correlation coefficients. It showed that Reg3b and Reg3g were significantly associated with the probiotic genus including *Lactobacillus* by Spearman correlation analysis (Figure 8).

## 4 Discussion

The paradox between the increasing incidence and limited approaches in preventing and treating IBD leads to the urgent need to discover potential compounds showing effective and safe protection against colonic inflammation. Our findings demonstrated that 2′‐FL, the most abundant HMO derived from human milk, significantly mitigated DSS‐induced intestinal inflammation, and the integration of colonic proteomics and fecal microbiota analysis indicated this beneficial effect was associated with the regulatory function on the gut microbiota and its interaction with the host immune response by 2′‐FL.

HMOs have considerable potential to alleviate the dysbiosis, preventing auto‐immune inflammatory damage, rescuing the mucosal barrier dysfunction in the previous studies (Paone et al. 2024; Rousseaux et al. 2021; Schönknecht et al. 2023). These biological functions of HMOs showed overlapping with the pathological changes in the etiology of clinical IBD. In the present study, the colonic proteomics and GO enrichment analysis suggested the involvement of 2′‐FL in various pathways, especially the two biological modules including *acute‐phase response* and *response to bacterium*. This result was in line with our findings showing 2′‐FL capable of mitigating intestinal morphological damage and dysbiosis caused by acute inflammation. Haptoglobin, a critical DEP that was significantly elevated in DSS‐induced colitis and reduced by 2′‐FL administration in the present research, is an acute phase protein with antimicrobial activity. Haptoglobin can be upregulated attending stimulation of cytokines such as IL‐1 and IL‐6 (Yang et al. 2016). It binds to free Hemoglobin produced by hemolysis, and prevents damage caused by oxidative stress (Motooka et al. 2021). And UC and CD patients exhibit high expression of haptoglobin (Soomro et al. 2021). It was assumed that compensatory upregulation of haptoglobin during the acute‐phase inflammatory response indicated intestinal immune reactions in response to activated inflammation. Also, we found that expression of serpina3n was significantly altered in IBD and interfered by 2′‐FL. Previous studies have also found that serpina3n, an inhibitor of NE release from T lymphocytes, is not only increased in serum and fecal expression of serpina3n in IBD patients, but also highly expressed in DSS‐induced colitis animals as well as in *Citrobacter rodentium* ‐stimulated IEC cells, which was associated with the inhibitory effects of serpina3n on tissue damage induced by extracellular neutrophil elastase (Barry et al. 2020; Ho et al. 2021). To date, handful evidences have revealed potential immunoregulatory targets of 2′‐FL in protecting against experimental IBD, our results indicated that haptoglobin and serpina3n were involved in the pharmacological function of 2′‐FL in attenuating colitis. Its underlying mechanisms remain to be fully elucidated.

In the DEPs correlation analysis, Arg2 and Cyp2e1 showed significant positive correlations. That meant the expression patterns of Arg2 and Cyp2e1 exhibit high coordination, indicating an intense regulation between them, in the condition of experimental colitis and 2′‐FL administration. This regulation was critical in revealing the protective function of 2′‐FL against intestinal inflammation. In the present study, Arg2 and Cyp2e1 were significantly increased in DSS‐induced colitis mice and decreased by 2′‐FL. The expression of Arg2 can be upregulated in response to intestinal oxidative stress in mice with DSS‐induced colitis and patients with ulcerative colitis (Coburn et al. 2016; Imazu et al. 2024). This upregulation is compensatory and protective, owing to the previous study showing that *Arg2*‐deficient (Arg2^−/−^) mice were more susceptible to oxidative stress and exhibited aggravated colonic inflammation characterized by goblet cell depletion with mucin 2 downregulation and mucosal barrier dysfunction, compared to wild‐type mice (Baier et al. 2020; Imazu et al. 2024; Zhao et al. 2010). The oxidative stress is implicated in the pathogenesis of IBD and is associated with overproduction of excessive reactive oxygen species (ROS) and other peroxide metabolites. Cytochromes P450 2E1 (CYP2E1), a main member of the cytochromes P450 super family that is responsible for the catabolic metabolism of many small molecules and hydrophobic compounds, also produces excessive amounts of ROS and leads to subsequent lipid peroxidation during the metabolism process (Makaro et al. 2025). The overexpression of intestinal CYP2E1 contributes to the disruption of barrier integrity and epithelial hyper‐permeability, partly due to the ROS‐releasing mechanism (Makaro et al. 2025; Mun et al. 2024) In this study, the abnormally elevated protein level of Cyp2e1, which was consistent with the clinical findings that the immunohistochemical expressions of Cyp2e1 in the enterocytes of colon tissues from patients with Crohn's disease or ulcerative colitis were elevated, compared to the controls (Plewka et al. 2014). The overexpression of Cyp2e1 possibly played a causal role in inducing Arg2 expression by triggering oxidative redox shift of the gut during intestinal inflammation. This was consistent with and explained the synchronized upregulation of Arg2 and Cyp2e1 in DSS‐induced colitis in the present study. Moreover, the significantly mitigated overexpression of Arg2 and Cyp2e1 by 2′‐FL suggested 2′‐FL exerted its protective function against intestinal inflammation through enhancing the epithelial barrier and mucosal function. This was in line with our morphological results showing 2′‐FL improved the integrity of intestinal tissue structure, particularly the epithelium layer.

One perspective that is gradually recognized is that 2′‐FL repairs the intestinal barrier damage, ameliorates intestinal permeability and integrity, and effectively attenuates the intestinal inflammation (Yao et al. 2022). Even though altered intestinal barrier homeostasis is considered the pathogenesis in intestinal inflammation, there are still substantial gaps in the underlying mechanism of barrier impairment. In our study, two intestinal barrier‐associated proteins, Reg3b and Reg3g, were found to participate in the protection of 2′‐FL in experimental IBD. Functioning as antimicrobial peptides, Reg3b and Reg3g express in response to the conditions of STAT3 hyperactivation as well as the upregulation of inflammatory cytokines such as IL‐18 and IL‐22 in intestinal epithelial cells during IBD. The pathological upregulation of Reg3b and Reg3g arises from the intestinal barrier damage and perpetuates STAT3/Akt self‐amplifying activation. This feed‐forward regulatory circuit finally facilitates the crypt cell proliferation and intestinal repair (Danne et al. 2024; Ratsimandresy et al. 2017; Xu et al. 2019). Our results indicated that 2′‐FL was likely to improve the repair of damaged intestinal mucosal barriers by upregulating Reg3b and Reg3g expression. Given that the expression of Reg3b and Reg3g is sensitive to the existence of special bacteria including *Escherichia‐Shigella*, *Listeria monocytogenes* , *Enterococcus*, and *Salmonella enteritidis* (Loonen et al. 2014; Tao et al. 2020; Wang et al. 2016), and that gut microbiota serves as the indispensable factor in maintaining homeostasis of the intestinal mucosal barrier, we subsequently performed 16S rRNA sequencing on cecal fecal in groups of mice and conducted a correlation analysis between the identified differential microbiota and DEPs. The correlation results revealed the close relation between Reg3g/Reg3b and *Lactobacillus* after the administration of 2′‐FL. *Lactobacillus*, the most essential probiotic bacteria of the gut microbiota, is known to be able to restore gastrointestinal barrier function (Rastogi and Singh 2022). It was suggested that the expression of Reg3g and Reg3b was modulated by microbiota and involved the protection of 2′‐FL in experimental IBD. A previous study has revealed that the inhibition of *Clostridia* led to a decrease in the levels of Reg3b and propionate, one of the predominant SCFAs produced by gut microbiota. The study demonstrated that *Clostridia* maintain the intestinal mucosal homeostasis through the propionate‐Reg3b axis (Bajic et al. 2020). In addition, clinical investigations have identified the enrichment of *Peptostreptococcaceae* and its inverse correlation with mucosal Reg3g expressions in UC patients, suggesting potential crosstalk between pathogen expansion and impaired antimicrobial defense barriers (Jalanka et al. 2020). It is worth mentioning that the regulation of Reg3 in the gut shows dual dynamic effects. On one hand, Reg3g and Reg3b are recognized as the righteous agents in eliminating pathogenic bacteria and protecting the intestinal tract. On the other hand, Reg3 overexpression leads to the reduction of probiotic bacteria abundance. Despite the fact that the role of Reg3 exerting prebiotic or opposite actions is largely unknown, growing evidence has demonstrated that Reg3 hyperexpression during acute IBD inflammation depletes intestinal *Enterococcus faecium* (a beneficial commensal symbiont) and thereafter blocks Nod2‐mediated anti‐inflammatory signaling (He and Zhou 2023; Jang et al. 2023). These evidences support Reg3 acting as a portative role in maintaining the immune‐homeostasis in the IBD condition, and the restoration of Reg3 levels by 2′‐FL indicated the relief of the inflammatory process and attenuation of dysbiosis in the colon of colitis mice in response to 2′‐FL treatment.

Increasing evidence indicates that modulating gut microbiota is a promising strategy to counteract IBD inflammation. In our study, DEPs within specific biological gene sets such as *response to bacterium*, *positive regulation of inflammatory response*, and *cellular response to reactive oxygen species* were upregulated in response to DSS, while 2′‐FL reversed these upregulations. This led to the indication that the gut microbiota was critically involved in the protective mechanisms of 2′‐FL in intestinal inflammation. We found that the relative abundance of the *Lachnospiraceae_NK4A136_group* was reduced, and *Escherichia‐Shigella* was increased in the DSS group compared to the controls. These findings were consistent with previous evidence showing that IBD exhibited a characteristic microbial variation marked by significant reduction in *Firmicutes* abundance accompanied by proportional elevation of *Proteobacteria* levels (Zhou et al. 2022). *Proteobacteria*, encompassing *Escherichia coli* , *Salmonella*, *Escherichia‐Shigella*, and other subtypes, enhance intestinal barrier permeability, induce immune dysregulation, and perpetuate inflammatory responses within the gut microenvironment (Mirsepasi‐Lauridsen et al. 2019). In our present study, quantitative microbiota analysis revealed dysbiosis characterized by depletion of probiotics concurrent with expansion of potentially pro‐inflammatory pathobionts (including *Escherichia‐shigella*, and *Helicobacter*) in the DSS group. This dysbiosis shift was significantly rescued by 2′‐FL treatment, as indicated by raised abundance of *Lachnospiraceae_NK4A136_group* and reduced abundance of *Escherichia‐Shigella* and *Bacteroides*, which was in line with the innate probiotic effects of 2′‐FL and demonstrated *Lachnospiraceae_NK4A136_group, Escherichia‐Shigella*, and *Bacteroides* as specific regulated bacterial genera by 2′‐FL in the pathological condition of colonic inflammation.

In the present study, label‐free quantitative proteomics combined with 16S rRNA sequencing integrated multi‐omics data at the holistic level, promoted the anchoring of pivotal molecules, and revealed novel protection mechanisms of 2′‐FL against experimental IBD in mice. 2′‐FL attenuated dysbiosis and showed modulation of the expressions of Reg3g and Reg3b, the functional molecules in *response to* the *bacterium* module. It down‐regulated haptoglobin and serpina3n, proteins involved in the *acute phase response*. Also, the expression of Arg2 showed a significant positive association with that of the pro‐oxidant indicator Cyp2e1. 2′‐FL significantly mitigated the overexpression of Arg2 and Cyp2e1, suggesting the protective capability of 2′‐FL in enhancing the epithelial barrier and mucosal homeostasis. Furthermore, administration of 2′‐FL showed potent regulatory effects on the gut microbiota environment, specifically curtailing the overgrowth of pathogenic *Proteobacteria* while restoring the abundance of the prebiotic bacterial genus *Lachnospiraceae_NK4A136_group* to homeostatic levels. Collectively, 2′‐FL provides protection against inflammatory damage via regulating gut microbiota and its interaction with host immunity in response to bacteria in experimental IBD, and functions as a potential natural compound in interfering with IBD.

## Author Contributions

**Jiamin Dong:** conceptualization (equal), data curation (equal), formal analysis (equal), investigation (equal), writing – original draft (equal), writing – review and editing (equal). **Minyan Qian:** formal analysis (equal), investigation (equal). **Dong Zhou:** conceptualization (equal), writing – review and editing (equal). **Aoshuang Zhu:** formal analysis (equal), investigation (equal). **Wenting Zhang:** conceptualization (equal), data curation (equal), formal analysis (equal), investigation (equal), writing – original draft (equal), writing – review and editing (equal).

## Ethics Statement

The experimental protocols involving animals were conducted with the approval of the Ethical Committee of Changzhou Children's Hospital Affiliated to Nantong University (Changzhou, China).

## Consent

The authors have nothing to report.

## Conflicts of Interest

The authors declare no conflicts of interest.

## Supporting information


**Table S1.** A list of 158 overlapped DEPs in two comparisons (DSS vs. control and DSS + 2′‐FL vs. DSS).

## Data Availability

All data relevant to the study are included in the article or uploaded as Supporting Information.
