# Supplementary material for: 2′‐Fucosyllactose Modulates the Intestinal Immune Response to Gut Microbiota and Buffers Experimental Colitis in Mice: An Integrating Investigation of Colonic Proteomics and Gut Microbiota Analysis
Source: Food Sci Nutr. 2025 Jun 10;13(6):e70418. doi: 10.1002/fsn3.70418 (PMC12152263; doi:10.1002/fsn3.70418)
Supplement: Supplementary file 1 — Table S1. A list of 158 overlapped DEPs in two comparisons (DSS vs. control and DSS + 2′‐FL vs. DSS). [file FSN3-13-e70418-s001.docx]

**2’-fucosyllactose modulates the intestinal immune response to gut microbiota and buffers experimental colitis in mice: an integrating investigation of colonic proteomics and gut microbiota analysis**

Jiamin Dong, Minyan Qian, Dong Zhou, Aoshuang Zhu, Wenting Zhang

**Supplementary Data of Contents**

Supplementary Data Table 1

**Supplementary Table 1** A list of 158 overlapped DEPs in two comparisons

(DSS vs. control and DSS+2’-FL vs. DSS)

| **No.** | **Gene Symbol** | **Description** | **Fold change** DSS/control | **Fold change** DSS+2’-FL/DSS |
| --- | --- | --- | --- | --- |
| 1 | Trim37 | E3 ubiquitin-protein ligase TRIM37 | 1489.7288 | 0.0001 |
| 2 | Hmgn1 | Non-histone chromosomal protein HMG-14 | 335.1651 | 0.0006 |
| 3 | Mt1 | Metallothionein-1 | 8865.7564 | 0.0006 |
| 4 | Rictor | Rapamycin-insensitive companion of mTOR | 733.8829 | 0.0014 |
| 5 | Ralgds | Ral guanine nucleotide dissociation stimulator | 155.3909 | 0.0015 |
| 6 | Saa2 | Serum amyloid A-2 protein | 1516.5969 | 0.0021 |
| 7 | Nhlrc3 | NHL repeat-containing protein 3 | 443.3247 | 0.0023 |
| 8 | Mt3 | Metallothionein-3 | 390.4294 | 0.0026 |
| 9 | Arg2 | Arginase-2, mitochondrial | 2.5062 | 0.0026 |
| 10 | Glmp | Glycosylated lysosomal membrane protein | 96.3136 | 0.0032 |
| 11 | Cyp2e1 | Cytochrome P450 2E1 | 2.3165 | 0.0032 |
| 12 | L3hypdh | Trans-L-3-hydroxyproline dehydratase | 29.9728 | 0.0040 |
| 13 | Fnbp1l | Formin-binding protein 1-like | 25.1090 | 0.0050 |
| 14 | Saa3 | Serum amyloid A-3 protein | 91.3522 | 0.0051 |
| 15 | Ier3ip1 | Immediate early response 3-interacting protein 1 | 139.2504 | 0.0066 |
| 16 | Timp2 | Metalloproteinase inhibitor 2 | 139.4914 | 0.0075 |
| 17 | Pnpla2 | Patatin-like phospholipase domain-containing protein 2 | 133.7417 | 0.0082 |
| 18 | Rbbp5 | Retinoblastoma-binding protein 5 | 1733.5359 | 0.0082 |
| 19 | Cd47 | Leukocyte surface antigen CD47 | 148.4359 | 0.0083 |
| 20 | Tlr3 | Toll-like receptor 3 | 34.3805 | 0.0114 |
| 21 | Mmab | Corrinoid adenosyltransferase MMAB | 31.9411 | 0.0130 |
| 22 | Phyh | Phytanoyl-CoA dioxygenase, peroxisomal | 3.0027 | 0.0132 |
| 23 | Rwdd4 | RWD domain-containing protein 4 | 227.1161 | 0.0151 |
| 24 | Pcsk1 | Neuroendocrine convertase 1 | 21.2582 | 0.0163 |
| 25 | Hmcn2 | Hemicentin-2 | 118.7732 | 0.0193 |
| 26 | Pdgfrb | Platelet-derived growth factor receptor beta | 114.4745 | 0.0261 |
| 27 | Abcb6 | ATP-binding cassette sub-family B member 6 | 37.3447 | 0.0264 |
| 28 | Reps1 | RalBP1-associated Eps domain-containing protein 1 | 39.3881 | 0.0268 |
| 29 | Stk3 | Serine/threonine-protein kinase 3 | 32.8737 | 0.0272 |
| 30 | Cntn1 | Contactin-1 | 33.8300 | 0.0321 |
| 31 | Rasa4 | Ras GTPase-activating protein 4 | 31.5357 | 0.0332 |
| 32 | Ly75 | Lymphocyte antigen 75 | 90.5454 | 0.0356 |
| 33 | Commd4 | COMM domain-containing protein 4 | 70.9090 | 0.0404 |
| 34 | Hp | Haptoglobin | 188.3763 | 0.1556 |
| 35 | Serpina3n | Serine protease inhibitor A3N | 18.0207 | 0.2874 |
| 36 | Mep1b | Meprin A subunit beta | 4.6062 | 0.3803 |
| 37 | Prom1 | Prominin-1 | 2.0323 | 0.3886 |
| 38 | Phyhd1 | Phytanoyl-CoA dioxygenase domain-containing protein 1 | 4.3691 | 0.3970 |
| 39 | Smpdl3a | Cyclic GMP-AMP phosphodiesterase SMPDL3A | 2.4230 | 0.4147 |
| 40 | St3gal6 | Type 2 lactosamine alpha-2,3-sialyltransferase | 0.2910 | 2.0609 |
| 41 | Nucks1 | Nuclear ubiquitous casein and cyclin-dependent kinase substrate 1 | 0.4993 | 2.0743 |
| 42 | Rsrc2 | Arginine/serine-rich coiled-coil protein 2 | 0.4248 | 2.4847 |
| 43 | Slc26a3 | Chloride anion exchanger | 0.2719 | 2.7060 |
| 44 | Cdk2 | Cyclin-dependent kinase 2 | 0.4515 | 3.0337 |
| 45 | Ubap2 | Ubiquitin-associated protein 2 | 0.4254 | 3.0837 |
| 46 | Clca1 | Calcium-activated chloride channel regulator 1 | 0.1512 | 3.2179 |
| 47 | Capn13 | Calpain-13 | 0.2864 | 3.3252 |
| 48 | B3gnt7 | UDP-GlcNAc:betaGal beta-1,3-N-acetylglucosaminyltransferase 7 | 0.2884 | 3.3445 |
| 49 | Fads2 | Acyl-CoA 6-desaturase | 0.4985 | 3.3952 |
| 50 | Ighg3 | Ig gamma-3 chain C region | 0.2568 | 3.5717 |
| 51 | Smc4 | Structural maintenance of chromosomes protein 4 | 0.4336 | 3.6360 |
| 52 | Ppat | Amidophosphoribosyltransferase | 0.3756 | 3.9642 |
| 53 | Ncapd2 | Condensin complex subunit 1 | 0.4070 | 4.9554 |
| 54 | Lv1a | Ig lambda-1 chain V region | 0.4075 | 6.8359 |
| 55 | Top2a | DNA topoisomerase 2-alpha | 0.2788 | 7.5099 |
| 56 | Atp12a | Potassium-transporting ATPase alpha chain 2 | 0.2118 | 12.7332 |
| 57 | Mptx1 | Mucosal pentraxin | 0.0105 | 18.9112 |
| 58 | Snta1 | Alpha-1-syntrophin | 0.0573 | 21.4841 |
| 59 | Inf2 | Inverted formin-2 | 0.0659 | 22.4395 |
| 60 | Fam241a | Uncharacterized protein FAM241A | 0.0386 | 23.6925 |
| 61 | Vps37b | Vacuolar protein sorting-associated protein 37B | 0.0416 | 25.4532 |
| 62 | Trmt5 | tRNA (guanine(37)-N(1))-methyltransferase | 0.0461 | 26.4323 |
| 63 | Nipbl | Nipped-B-like protein | 0.0341 | 27.3789 |
| 64 | Pcm1 | Pericentriolar material 1 protein | 0.0297 | 27.9663 |
| 65 | Ehf | ETS homologous factor | 0.0293 | 28.5325 |
| 66 | Leo1 | RNA polymerase-associated protein LEO1 | 0.0348 | 29.7408 |
| 67 | Naa20 | N-alpha-acetyltransferase 20 | 0.0360 | 30.8080 |
| 68 | Islr | Immunoglobulin superfamily containing leucine-rich repeat protein | 0.0422 | 31.6702 |
| 69 | Ndc1 | Nucleoporin NDC1 | 0.0395 | 32.9438 |
| 70 | Fut4 | Alpha-(1,3)-fucosyltransferase 4 | 0.0197 | 33.6997 |
| 71 | Slain2 | SLAIN motif-containing protein 2 | 0.0245 | 34.9162 |
| 72 | Chlsn | Cholesin | 0.0382 | 34.9485 |
| 73 | Retreg3 | Reticulophagy regulator 3 | 0.0231 | 35.9461 |
| 74 | Parg | Poly(ADP-ribose) glycohydrolase | 0.0395 | 37.6617 |
| 75 | Eed | Polycomb protein EED | 0.0348 | 38.7081 |
| 76 | Pick1 | PRKCA-binding protein | 0.0112 | 39.5937 |
| 77 | Urah | 5-hydroxyisourate hydrolase | 0.0311 | 39.6804 |
| 78 | Mob3a | MOB kinase activator 3A | 0.0255 | 39.7854 |
| 79 | Rnf121 | E3 ubiquitin ligase Rnf121 | 0.0193 | 41.2166 |
| 80 | Dcaf11 | DDB1- and CUL4-associated factor 11 | 0.0148 | 42.0993 |
| 81 | Vamp7 | Vesicle-associated membrane protein 7 | 0.0266 | 42.8985 |
| 82 | Max | Protein max | 0.0411 | 43.1200 |
| 83 | Zfand6 | AN1-type zinc finger protein 6 | 0.0162 | 43.9079 |
| 84 | Timm29 | Mitochondrial import inner membrane translocase subunit Tim29 | 0.0256 | 44.3073 |
| 85 | Slx9 | Ribosome biogenesis protein SLX9 homolog | 0.0398 | 44.5876 |
| 86 | Exog | Nuclease EXOG, mitochondrial | 0.0113 | 45.6145 |
| 87 | Cox7a2l | Cytochrome c oxidase subunit 7A2-like, mitochondrial | 0.0235 | 46.6350 |
| 88 | Mmgt1 | ER membrane protein complex subunit 5 | 0.0194 | 46.7502 |
| 89 | Gpkow | G-patch domain and KOW motifs-containing protein | 0.0333 | 47.7414 |
| 90 | Tapt1 | Transmembrane anterior posterior transformation protein 1 | 0.0193 | 50.0466 |
| 91 | Mark3 | MAP/microtubule affinity-regulating kinase 3 | 0.0216 | 51.3805 |
| 92 | Rfc3 | Replication factor C subunit 3 | 0.0453 | 51.6314 |
| 93 | Mphosph10 | U3 small nucleolar ribonucleoprotein protein MPP10 | 0.0422 | 54.0392 |
| 94 | Arhgef5 | Rho guanine nucleotide exchange factor 5 | 0.0218 | 55.4562 |
| 95 | Znf622 | Cytoplasmic 60S subunit biogenesis factor ZNF622 | 0.0253 | 55.9201 |
| 96 | Scaf1 | Splicing factor, arginine/serine-rich 19 | 0.0295 | 56.4335 |
| 97 | Nudcd3 | NudC domain-containing protein 3 | 0.0277 | 57.5159 |
| 98 | Klhdc4 | Kelch domain-containing protein 4 | 0.0189 | 58.1184 |
| 99 | Heatr5a | HEAT repeat-containing protein 5A | 0.0268 | 59.6221 |
| 100 | Rptor | Regulatory-associated protein of mTOR | 0.0184 | 60.5730 |
| 101 | Gal3st2 | Galactose-3-O-sulfotransferase 2 | 0.0204 | 61.5065 |
| 102 | Utp15 | U3 small nucleolar RNA-associated protein 15 homolog | 0.0294 | 61.6759 |
| 103 | Znf706 | Zinc finger protein 706 | 0.0159 | 65.8104 |
| 104 | Lsm14b | Protein LSM14 homolog B | 0.0104 | 67.1225 |
| 105 | Lig1 | DNA ligase 1 | 0.0260 | 69.7962 |
| 106 | Crocc | Rootletin | 0.0133 | 71.5744 |
| 107 | Ngdn | Neuroguidin | 0.0209 | 73.3747 |
| 108 | Ints4 | Integrator complex subunit 4 | 0.0187 | 74.1469 |
| 109 | Tk1 | Thymidine kinase, cytosolic | 0.0207 | 74.9370 |
| 110 | Pno1 | RNA-binding protein PNO1 | 0.0302 | 76.1021 |
| 111 | Zc2hc1a | Zinc finger C2HC domain-containing protein 1A | 0.0168 | 76.8390 |
| 112 | Tut7 | Terminal uridylyltransferase 7 | 0.0131 | 77.5811 |
| 113 | Znf830 | Zinc finger protein 830 | 0.0130 | 84.9559 |
| 114 | Rtf2 | Replication termination factor 2 | 0.0118 | 85.9743 |
| 115 | Derl1 | Derlin-1 | 0.0116 | 90.1259 |
| 116 | Nde1 | Nuclear distribution protein nudE homolog 1 | 0.0111 | 91.5145 |
| 117 | Cnot10 | CCR4-NOT transcription complex subunit 10 | 0.0109 | 91.6727 |
| 118 | Trir | Telomerase RNA component interacting RNase | 0.0137 | 93.5211 |
| 119 | Trappc2 | Trafficking protein particle complex subunit 2 | 0.0117 | 101.9844 |
| 120 | Chmp5 | Charged multivesicular body protein 5 | 0.0085 | 109.7368 |
| 121 | Maged1 | Melanoma-associated antigen D1 | 0.0145 | 112.2071 |
| 122 | Specc1l | Cytospin-A | 0.0082 | 113.3525 |
| 123 | Sbf2 | Myotubularin-related protein 13 | 0.0081 | 113.6792 |
| 124 | Trafd1 | TRAF-type zinc finger domain-containing protein 1 | 0.0108 | 117.1699 |
| 125 | Spats2l | SPATS2-like protein | 0.0129 | 119.1329 |
| 126 | Clic6 | Chloride intracellular channel protein 6 | 0.0139 | 120.6824 |
| 127 | Elmod2 | ELMO domain-containing protein 2 | 0.0094 | 126.0334 |
| 128 | Ftsj3 | pre-rRNA 2'-O-ribose RNA methyltransferase FTSJ3 | 0.0126 | 127.5108 |
| 129 | Sdhd | Succinate dehydrogenase [ubiquinone] cytochrome b small subunit, mitochondrial | 0.0036 | 140.4429 |
| 130 | Mepce | 7SK snRNA methylphosphate capping enzyme | 0.0053 | 141.6446 |
| 131 | Wdr75 | WD repeat-containing protein 75 | 0.0108 | 174.4104 |
| 132 | Nle1 | Notchless protein homolog 1 | 0.0056 | 179.3769 |
| 133 | Serpina6 | Corticosteroid-binding globulin | 0.0124 | 191.9954 |
| 134 | Znrd2 | Protein ZNRD2 | 0.0085 | 193.5510 |
| 135 | Brd3 | Bromodomain-containing protein 3 | 0.0049 | 198.0079 |
| 136 | Cps1 | Carbamoyl-phosphate synthase [ammonia], mitochondrial | 0.0050 | 200.3474 |
| 137 | Reep6 | Receptor expression-enhancing protein 6 | 0.0060 | 205.7816 |
| 138 | Ppm1h | Protein phosphatase 1H | 0.0033 | 226.6430 |
| 139 | Tmem70 | Transmembrane protein 70, mitochondrial | 0.0040 | 232.4560 |
| 140 | Ddx50 | ATP-dependent RNA helicase DDX50 | 0.0044 | 236.9867 |
| 141 | Magi1 | Membrane-associated guanylate kinase, WW and PDZ domain-containing protein 1 | 0.0040 | 300.3119 |
| 142 | Sbno1 | Protein strawberry notch homolog 1 | 0.0041 | 312.9577 |
| 143 | Nol6 | Nucleolar protein 6 | 0.0053 | 321.4677 |
| 144 | Eef1akmt1 | EEF1A lysine methyltransferase 1 | 0.0029 | 322.3039 |
| 145 | Ddx56 | Probable ATP-dependent RNA helicase DDX56 | 0.0053 | 370.9338 |
| 146 | Plek2 | Pleckstrin-2 | 0.0046 | 445.0137 |
| 147 | Cops7b | COP9 signalosome complex subunit 7b | 0.0034 | 513.1063 |
| 148 | Ntaq1 | Protein N-terminal glutamine amidohydrolase | 0.0016 | 542.3604 |
| 149 | C15orf40 | UPF0235 protein C15orf40 homolog | 0.0019 | 581.4696 |
| 150 | Erc1 | ELKS/Rab6-interacting/CAST family member 1 | 0.0020 | 636.5879 |
| 151 | Ncaph | Condensin complex subunit 2 | 0.0025 | 675.8241 |
| 152 | Spink4 | Serine protease inhibitor Kazal-type 4 | 0.0010 | 755.2738 |
| 153 | Glrx2 | Glutaredoxin-2, mitochondrial | 0.0007 | 806.0793 |
| 154 | Kif4 | Chromosome-associated kinesin KIF4 | 0.0014 | 847.6859 |
| 155 | Ang4 | Angiogenin-4 | 0.0003 | 940.4712 |
| 156 | Ctse | Cathepsin E | 0.0020 | 1266.7710 |
| 157 | Tmem41b | Transmembrane protein 41B | 0.0006 | 1540.5417 |
| 158 | Pbk | Lymphokine-activated killer T-cell-originated protein kinase | 0.0002 | 5970.6157 |

Proteins which have fold change > 2 or < 0.5 and p value < 0.05 in the comparison were screened as DEPs.
